# Obesity Is Associated with Asymptomatic Vertebral Fractures: A Yakumo Study

**DOI:** 10.3390/jcm13072063

**Published:** 2024-04-02

**Authors:** Yuichi Miyairi, Hiroaki Nakashima, Sadayuki Ito, Naoki Segi, Jun Ouchida, Ryotaro Oishi, Ippei Yamauchi, Masaaki Machino, Taisuke Seki, Shinya Ishizuka, Yasuhiko Takegami, Yukiharu Hasegawa, Shiro Imagama

**Affiliations:** 1 Department of Orthopaedic Surgery, Nagoya University Graduate School of Medicine, Nagoya 466-8560, Japan; miyairi0317@gmail.com (Y.M.); sadaito@med.nagoya-u.ac.jp (S.I.); naoki.s.n@gmail.com (N.S.); orthochida@gmail.com (J.O.); ryo.oishi@gmail.com (R.O.); Yaip0411@yahoo.co.jp (I.Y.); shinyai@med.nagoya-u.ac.jp (S.I.); takegami@med.nagoya-u.ac.jp (Y.T.); imagama@med.nagoya-u.ac.jp (S.I.); 2 Department of Orthopedic Surgery, Meijo Hospital, Nagoya 460-0001, Japan; Masaki_Macchino_5445_2@yahoo.co.jp; 3 Department of Orthopedic Surgery, Aichi Medical University Medical Center, Nagakute 444-2148, Japan; taiseki@aichi-med-u.ac.jp; 4 Department of Rehabilitation, Kansai University of Welfare Science, Kashiwara 582-0026, Japan; hasegawa@tamateyama.ac.jp

**Keywords:** asymptomatic vertebral fractures, body fat percentage (BF%), knee osteoarthritis (KOA), painDETECT, resident health examination

## Abstract

**(1) Background:** Patients with primary vertebral fracture (VF) are at high risk of re-fracture and mortality. However, approximately two-thirds of patients with VFs receive minimal clinical attention. **(2) Methods:** The current study aimed to investigate the factors associated with asymptomatic VFs in middle-aged and elderly individuals who underwent resident health examinations. **(3) Results:** The current study included 217 participants aged > 50 years. VFs were diagnosed based on lateral radiographic images using Genant’s semiquantitative (SQ) method. The participants were divided into non-VF (N; SQ grade 0) and asymptomatic VF (F; SQ grades 1–3) groups. Data on body composition, blood tests, quality of life measures, and radiographic parameters were assessed. A total of 195 participants were included in the N group (mean age, 64.8 ± 7.8 years), and 22 were in the F group (mean age, 66.1 ± 7.9 years). The F group had a significantly higher body mass index (BMI), body fat percentage (BF%), and proportion of patients with knee osteoarthritis (KOA) than the N group. The F group had a significantly higher knee joint pain visual analog scale (VAS) score and painDETECT score than the N group. Logistic regression analysis showed that BF% was associated with asymptomatic VFs. **(4) Conclusions:** Middle-aged and elderly individuals with asymptomatic VF presented with high BMIs, BF%, and incidence of KOA.

## 1. Introduction

The prevalence of osteoporosis is increasing with the aging society [1,2]. Osteoporosis can cause fractures, leading to pain and motor dysfunction, which can be a major issue. Osteoporosis is a systemic, metabolic skeletal disease characterized by reduced bone quality and decreased bone mass with the destruction and deterioration of bone microstructure. This, in turn, induces a predisposition to bone fragility and overall decrease in bone strength, consequently leading to an increased risk of fracture [3]. Vertebral fractures (VFs) are among the most common types of osteoporotic fractures [4].

The diagnosis of VFs involves combining an evaluation of the patient’s age and medical history with a radiological examination. Post-traumatic back pain, reduction in height, and radiographic images are all basic diagnostic parameters. One of the conventional methods of VF identification in radiographs is the semiquantitative (SQ) evaluation method. VF evaluation using the SQ method is based on the reduction of vertebral height. The anterior, middle, and posterior heights of the vertebrae are evaluated by visual inspection. The vertebrae are then classified as normal (grade 0), mildly deformed (grade 1, 20–25% reduction in one of the three heights and 10–20% reduction in area), moderately deformed (grade 2, 25–40% reduction in any one height and 20–40% reduction in area), and highly deformed (grade 3, height and area (>40% reduction in height and area), respectively. Because there is no consensus on the exact definition of a VF, it may sometimes be difficult to discriminate the prevalent VF from a normal variant of vertebral shape or from a vertebral deformation that may have occurred long ago, especially in mild cases. An osteoporotic VF appears as an alteration in the shape and size of the vertebral body, associated or not to vertebral height loss, resulting as a wedge, end-plate (mono-or biconcave), or collapse vertebral deformity [5].

DXA measurement is useful in determining the extent of osteoporosis and evaluating bone density to allow for the prediction of future fractures. Bone strength can be quantified using bone mineral density (BMD) and/or bone quality. While tools exist to accurately quantify BMD, the accurate measurement of bone quality within the clinical setting remains elusive. Thus, the measurement of BMD is the most effective method for determining the rate of bone loss and monitoring disease progression [6]. Undernutrition, particularly protein undernutrition, contributes to the occurrence of osteoporotic fracture by lowering bone mass and altering muscle strength [7]. The Geriatric Nutritional Risk Index (GNRI) is a nutritional screening index that was proposed to assess the nutrition-related risk originally for hospitalized elderly persons by Bouillanne et al. [8]. The GNRI is a simple and objective index, allowing clinicians to assess patients readily based on height, weight, and serum albumin level. The diagnosis of VF requires a multifaceted diagnosis that includes osteoporosis. However, the majority of osteoporotic VFs are asymptomatic and occur in the absence of specific trauma. Even these mild VFs can have clinical consequences for the patient because of the increased, approximately 5-fold, risk of future fractures that may be symptomatic [5]. In summary, patients with their first VF are at high risk of re-fracture [9]. Multiple VFs are associated with an increased mortality rate and impaired quality of life. Therefore, early therapeutic intervention is required. However, numerous patients with VFs remain undiagnosed and untreated because they have few or no symptoms. VFs without symptoms in daily life are referred to as asymptomatic or morphometric VFs [10].

VFs are generally associated with a decreased quality of life (QOL) [11]. In the acute phase, these types of fractures commonly heal with kyphotic deformity. However, some VFs become nonunion [12]. In addition, kyphotic deformity is more advanced in secondary multivertebral fractures compared with single VFs. Kyphotic deformity and nonunion often result in decreased mobility and chronic pain, thereby limiting activities of daily living [13]. Hence, initial VFs are often overlooked because of the absence or paucity of symptoms, and secondary VFs frequently lead to chronic back pain and reduced QOL. Decreased activity associated with VFs not only causes pain but also increases loneliness. Moreover, it can lead to depression [14], which has a significant impact on the QOL and longevity of elderly individuals.

VFs may cause changes in spinal alignment, and vertebral fractures may affect the lower extremities, including the knee. The relationship between vertebral fractures and knee osteoarthritis (KOA) has been reported previously. In addition, despite high systemic BMD, KOA is positively associated with VFs [15].

There are several cases of asymptomatic VFs upon the initial fracture. Few studies have evaluated factors associated with asymptomatic VFs. The characteristics of patients with asymptomatic VFs are not clear. Furthermore, their QOL is unknown.

The Yakumo study commenced in 1983 and established one of the cancer cohorts, and musculoskeletal disorders were also introduced as a study subject in 1989. The study subjects were healthy volunteers who participated in a health checkup that was held annually in the town of Yakumo in Hokkaido, Japan, and was supported by the local government. Orthopedic and physical function and neurological examinations were conducted by orthopedic joint specialists, physical therapists, and spine specialists.

The purpose of this study is to compare the differences between healthy volunteers without VFs and those with asymptomatic VFs and to evaluate the characteristics of asymptomatic VFs. For this purpose, the visual analog scale (VAS) for pain associated with vertebral fractures and knee osteoarthritis, the painDETECT for neuropathic pain [16], and the SF36 and EQ5D-5L for the association between anxiety/depression and VF were used to assess the quality of life scores in detail [17,18]. This study may help in efforts to reduce secondary VFs in daily practice.

## 2. Materials and Methods

### 2.1. Study Participants

The participants underwent a municipal-supported health checkup in the town of Yakumo in 2019. The research protocol was approved by the Human Research Ethics Committee and Institutional Review Board of our university. All participants provided written informed consent prior to participation. The research procedure was conducted in accordance with the principles of the Declaration of Helsinki.

The purpose of this study was to identify factors that contribute to the early detection of asymptomatic vertebral fractures. Therefore, we compared a group of asymptomatic VFs with a healthy group without VFs. We hypothesized that factors associated with vertebral fractures, such as osteoporosis, may be equally relevant to asymptomatic vertebral fractures. Therefore, we evaluated tests related to these issues.

The physical examinations included voluntary orthopedic and physical function tests, internal examinations, psychological tests, and a health-related QOL survey (The 36-Item Short-Form Survey) [19,20]. The severity of low back pain (LBP), leg pain, and knee joint pain in the last week on a visual analog scale (VAS; 0–100 mm) were examined as factors associated with vertebral fractures and knee osteoarthritis. PainDETECT was used for the detection of neuropathic pain (NeP) components in chronic low back pain participants [15]. The association between frailty and VFs was examined as described below. The SF36 (Japanese ver. 2.0) was used for the evaluation of QOL [16]. The eight scales and two summary measures of the SF36 (physical component summary [PCS] and mental component summary [MCS]) were evaluated. Subjects answered questions by themselves but with support if required.

**Figure 1:**
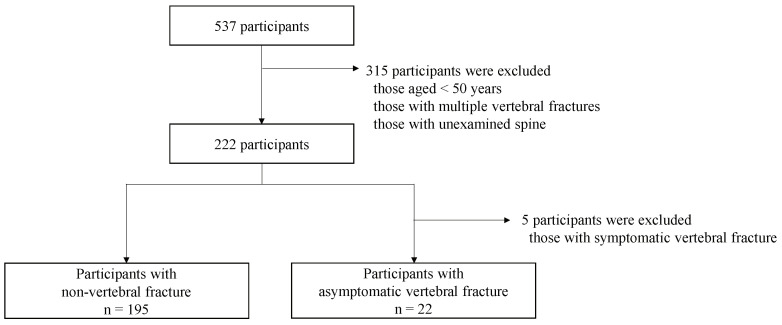
In total, 217 participants who underwent a resident health examination in 2019 were evaluated.

This study included all participants who underwent bioelectrical impedance analysis (BIA) and fasting blood glucose test. The exclusion criteria were as follows: participants aged < 50 years and those with VF with back pain, multiple VFs, unexamined spine, symptomatic VF, a history of spine or joint surgery, severe knee injury, severe mental illness, diabetes, kidney or heart disease, non-fasting, severe walking or standing impairment, and compromised central or peripheral nervous system. Of 537 participants who underwent health checkups, 217 met the inclusion criteria (Figure 1).

Table 1 shows the characteristics of the participants. There were 89 men and 128 women, with an average age of 64.9 ± 7.9 years (50–87 years). In total, 195 (82 men, 113 women) and 22 (7 men, 15 women) participants were included in the N and F groups, respectively. The fracture levels were T9:1, T12:4, L1:4, L2:7, L3:4, and L4:2. The fracture types were SQ1:9, SQ2:10, and SQ3:2.

### 2.2. Examination of Motor Function

Grip strength in the standing position was measured once for each hand using a hand-grip dynamometer (Toei Light Co., Ltd., Saitama, Japan). The mean value was used for analyses [21].

### 2.3. Bioelectrical Impedance Analysis (BIA)

BIA was used to analyze the body composition of the participants. The participants underwent the BIA on an empty stomach. The conditions of BIA measurement, such as consumption of food and beverages, were similar to those reported earlier [22]. The Inbody 770 BIA device (Inbody Co., Ltd., Seoul, Republic of Korea), which can differentiate fat, muscle, and bone tissues based on their electrical impedance, was used [20]. The participants grasped the handles of the analyzer, which have embedded electrodes, and stood on the platform with the soles of their feet in contact with the electrodes. There were two electrodes for each foot and hand. Anthropometric data, including height, weight, body mass index (BMI), body fat percentage (BF%), and appendicular skeletal muscle mass index (SMI), were measured. The BMI was calculated using the following formula: weight (kg) divided by height (m^2^). The muscle mass of each limb and BF% were automatically calculated based on BIA using the Inbody 770 BIA device. The SMI was calculated using the following formula: SMI = appendicular skeletal muscle mass (kg) divided by height (m^2^) [23].

### 2.4. Blood Sample Assessment

During the checkup, fasting blood samples were collected via venipuncture and centrifuged within 1 h of sampling. Serum samples were stored at −80 °C until the assay was performed. Routine biochemical analyses were performed in the laboratory of Yakumo Town Hospital. The purpose of the biochemical tests was to investigate whether nutritional status affects osteoporosis and vertebral fractures. In this study, serum albumin, total cholesterol, triglyceride, and C-reactive protein levels, which are related to nutrition, were examined [23].

### 2.5. Radiological Assessment

Radiographic images were obtained under the following standard conditions: (1) while the participants were standing and staring straight ahead with hands on the clavicles, (2) acquisition of two views (lateral craniopelvic and lateral pelvic), and (3) a distance of 1.5 m between the X-ray tube and radiograph. Digitized radiographic images were transferred to a computer as Digital Imaging and Communications in Medicine data and evaluated using an imaging software (Surgimap Spine Version 2.3.1; Nemaris Inc., New York City, NY, USA). Radiographic images were taken by a routinely trained radiographer.

**Figure 2:**
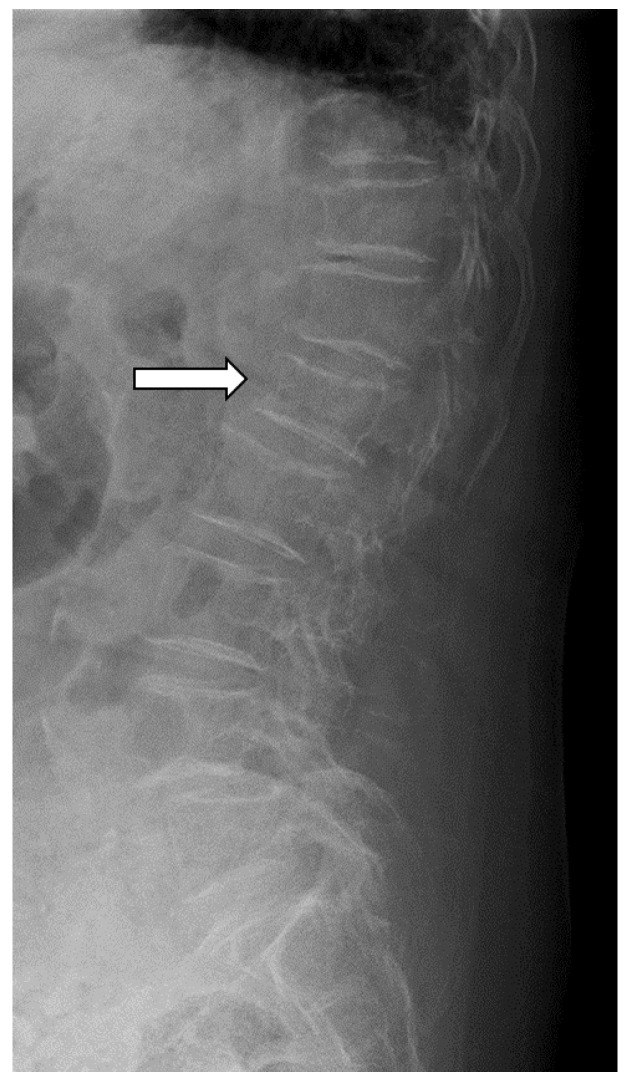
A 52-year-old female participant with L1 asymptomatic vertebral fractures (SQ grade 1). White arrows indicate mild compression of the first lumbar vertebra on the lateral lumbar spine X-ray image.

Genant’s semiquantitative method (SQ) was used to define the different types of VF (SQ ≥ 1): mild VF, SQ = 1; moderate VF, SQ = 2; and severe VF, SQ = 3. The participants were divided into non-VF (N; SQ grade 0) and asymptomatic VF (F; SQ grades 1–3) groups [24] (Figure 2).

VFs result in increased spinal inclination angle and imbalance. The result is a cause of low back pain and reduced quality of life. Additionally, the spinal global sagittal malalignment is compensated for at the lower extremity joints. To investigate these effects in asymptomatic vertebral fractures, spinal parameters and knee osteoarthritis were evaluated.

The parameters measured on spinal radiography were as follows: C2 slope (°), cervical lordosis (°), C2–C7 sagittal vertical axis (mm), T1 slope (°), thoracic kyphosis (°), thoracolumbar kyphosis (°), L4–S1 lordosis (°), lumbar lordosis (°), pelvic tilt, sacral slope (°), pelvic incidence (°), and pelvic incidence–lumbar lordosis (°). The global parameters measured radiographically included the sagittal vertical axis, cervicothoracic pelvic angle, C2 pelvic angle, and T1 pelvic angle [25].

Furthermore, the Kellgren and Lawrence (KL) radiographic classification system was used to diagnose knee osteoarthritis (KOA), with consideration of the following features: joint space narrowing, osteophyte formation on the joint margins or tibial spines, subchondral sclerosis, and bone-end deformation [26]. The KL classification was used to define KOA as follows: normal or suspected KOA, grade 0 or 1; KOA, grades 2–4.

### 2.6. Statistical Analyses

Continuous variables were expressed as mean ± standard deviation. The variables of the N group were compared to those of the F group using the Mann–Whitney U test. Meanwhile, the variables of the N group were compared to those of the F group using the chi-square test.

Logistic regression analysis was performed to evaluate factors associated with the asymptomatic vertebral fractures. Variables with a *p* value of <0.05 in the univariate analysis were included in the multivariate analysis. The N and F groups significantly differed in BMI, BF%, and KOA. Among them, the factors associated with obesity were examined as covariates for the risk factors of the F group in the logistic regression analysis. All statistical analyses were performed using the Statistical Package for the Social Sciences software version 26.0 for Windows (IBM Corp., Armonk, NY, USA). A *p* value of <0.05 was considered statistically significant.

## 3. Results

The purpose of this study was to identify factors that contribute to the early detection of asymptomatic vertebral fractures. Therefore, we compared a group of asymptomatic VFs with a healthy group without VFs.

The F group had a significantly higher BMIs (25.1 ± 2.9 vs. 23.8 ± 3.4 kg/m^2^, *p* = 0.031) and BF% (33.0% ± 7.9% vs. 29.2% ± 7.1%, *p* = 0.018) than the N group. The F group had a significantly higher proportion of participants with KOA than the N group (11/11 vs. 54/138, *p* = 0.035). The F group had a larger hip circumference than the N group (94.3 ± 5 cm vs. 92.1 ± 6.2 cm, *p* = 0.079). There were no significant differences between the N and F groups in terms of laboratory data (Table 1). Asymptomatic vertebral fractures were associated with obesity. However, there were no significant differences in osteoporosis, muscle strength, and nutritional indices.

In terms of radiographic parameters, the F group had a significantly higher proportion of participants with KOA (KL: grade > 2) than the N group (11/11 vs. 54/138, *p* = 0.035). There were no significant differences between the N and F groups in terms of spinal radiographic parameters. Asymptomatic VFs were a significant comorbidity of KOA. In asymptomatic VFs at a single vertebra, the spinal radiographic parameters did not change significantly (Table 2).

Obesity was defined as a BMI of ≥25 kg/m^2^ (Japan Society for the Study of Obesity, Inbody 770 BIA) and BF% of ≥28% (Inbody 770 BIA). In the logistic regression analysis, a larger BMI was a risk factor of asymptomatic VFs (Exp [B]: 3.091, 95% confidence interval: 1.100–8.684, *p* = 0.032) (Table 3).

In terms of QOL measurements, the F group had a significantly higher knee pain visual analog scale score (23.4 ± 19.7 vs. 13.5 ± 18.8, *p* = 0.014) and painDETECT score (5.7 ± 4.0 vs. 4.1 ± 4.4, *p* = 0.043) than the N group. There were no significant differences between the N and F groups in terms of the other QOL scores (SF-36 MCS, EQ5D-5L) related to anxiety and depression. Participants with asymptomatic VFs had a significantly higher level of knee pain and neuropathic pain (Table 4).

## 4. Discussion

This is the first large-scale study on asymptomatic VFs. The prevalence of osteoporotic VFs ranges from 9% to 26% worldwide, and its prevalence rate in Japan is 24% [9]. Two-thirds of patients with VF receive minimal clinical attention, and they are believed to have asymptomatic or morphometric VFs [11]. Patients with these types of fractures have few subjective symptoms. Thus, they may go undiagnosed and untreated. Based on previous reports, 16% of elderly individuals in Japan are likely to present with asymptomatic VFs. That is, 24% of elderly individuals in Japan have fractures, and two-thirds of them are asymptomatic. In the current study, the prevalence rate of asymptomatic VFs was 10% (22/227) in middle-aged and elderly participants. Furthermore, the prevalence rate in this research was similar to that of previous studies [9,10].

Initial VFs are often overlooked because of the absence or paucity of symptoms, and secondary VFs frequently lead to chronic back pain and reduced QOL. The purpose of this study is to reduce secondary VFs by early detection and therapeutic intervention of initial asymptomatic VFs. Therefore, we evaluated and clarified the characteristics of asymptomatic VFs by comparing them with healthy subjects. There are several reports about the risk factors of asymptomatic VFs. However, only a few studies have examined the association between asymptomatic VF and muscle mass, muscle strength, fat mass, and spinal alignment [27,28]. The current study showed that asymptomatic VFs were associated with BMI, BF%, knee pain VAS score, KOA, and painDETECT score in the univariate analysis. Moreover, a high BMI was a significant factor associated with asymptomatic VF in the multivariate analysis. Traditionally, a low BMI is related to an increased risk of fracture, and obesity is not considered a risk factor [29]. However, some studies have shown that obesity according to BF% is associated with an increased risk of fracture [30]. The risk of falls and a high vertebral load caused by weight gain may be a risk factor of VFs [30]. Moreover, recent studies have shown that obese women have a higher VF risk during standing, holding, and lifting because of higher loads from a greater body weight [31]. Thus, in some cases, individuals with higher BMIs are at risk of VF. The current study showed that a high BMI may be particularly related to asymptomatic VFs.

Interestingly, a higher BF% was also associated with asymptomatic VF. However, there was no significant difference in terms of BMD, which might be attributed to a higher bone marrow adipose tissue (BMAT). In addition, BMAT is inversely related to bone mass [32,33], which is also involved in VFs in elderly individuals [34]. One of the causes of this phenomenon is that bone marrow adipocytes and osteoblasts are derived from the same progenitor cell type. Hence, adipogenesis and osteoblastogenesis are two competing processes [35]. Furthermore, there was no decrease in BMD. However, bone weakness might be observed, which is associated with increased BMAT. If the BF% is greater, the BMAT is higher [36,37]. Increased body fat may be associated with a higher BMAT and worse bone quality. There was also no decrease in BMD. Nevertheless, poor bone quality might have caused VFs in the current study.

Spinal global sagittal malalignment is also associated with low back pain and decreased QOL [24]. In this study, there were no significant differences in alignment abnormalities in VFs. Since only one VF case was included and muscle weakness was not observed, early bone fusion and mild progression of kyphosis might have caused the earlier reduction or disappearance of low back pain. However, in elderly individuals with increased spinal inclination angle, forward-bent head, and sagittal imbalance, hip and knee flexion must be maintained to preserve their global sagittal alignment and to help them stand appropriately on their feet. Therefore, increased spinal tilt angle is related to a higher incidence of KOA. In addition, elderly individuals have difficulty maintaining sagittal balance because of spine inflexibility, which leads to sagittal imbalance. Spinal ROM and spinal tilt angle are also associated with KOA [38]. Static spinal alignment changes were not observed in the current study. However, changes in the dynamic gravity line during walking and other movements could have led to changes in knee load, thereby resulting in KOA. Alternatively, KOA might have originally existed, and changes in spinal loading associated with KOA could have led to fractures. KOA-related knee pain was more common in the F group, and this type of pain was associated with more severe neuropathic pain as identified using painDETECT. Further, it might have led to asymptomatic VFs of the spine due to imbalance during gait. In the current study, the primary cause, whether knee pain or asymptomatic VF, was not clear. Hence, further research should be conducted.

In summary, BMI, BF%, and KOA were associated with characteristics of asymptomatic VFs, and high BMI may be a risk factor. Obesity with increased BF% may lead to poor bone quality, and increased weight may cause vertebral fractures even with minor trauma. However, the patients might have had fewer pain symptoms and shorter pain periods because they maintained muscle strength to stabilize their trunks. Therefore, unlike painful vertebral fractures, asymptomatic VFs might not reflect mental QOL scores such as anxiety and depression. Although a single vertebral fracture does not result in spinal imbalance, unconscious lower extremity compensation may be associated with lower extremity pain. Conversely, KOA could have caused VF because of the strain on the spine. In addition, obesity has already been reported to be associated with KOA [39]. Therefore, asymptomatic VFs may not explain all causal relationships with obesity and KOA. Additional longitudinal studies are needed to establish these causal relationships. In any case, VFs should be considered in patients with high BMI and incidence of KOA, even for minor low back pain.

The current study had several limitations. First, the participants were middle-aged and elderly adults who lived in a relatively rural area, and many were employed in the agriculture or fishing sectors. Thus, there may be differences in lifestyle habits between the participants and people in an urban environment. Second, the participants in the study might have been more health-conscious than the general population based on their attendance at annual health examinations. Individuals who regularly participate in health checkups may have different characteristics or health profiles than those who do not attend such screenings. Third, this study did not compare asymptomatic and symptomatic VFs. The design of this study does not clarify all the mechanisms by which VFs are asymptomatic. Fourth, this was a cross-sectional, single-center study. In the future, longitudinal and multicenter collaborative research is needed to verify our findings.

## 5. Conclusions

We investigated the factors associated with asymptomatic vertebral fractures in community-dwelling middle-aged and older adults through a cross-sectional study. Middle-aged and elderly adults with asymptomatic vertebral fractures presented with high body fat percentages, body mass indices, and incidence of knee osteoarthritis. The results suggested that obesity, knee osteoarthritis, and asymptomatic vertebral fractures may each be closely related. Thus, these factors should be considered in future therapeutic interventions.

## Figures and Tables

**Table 1:** Demographic characteristics, physical fitness data, and laboratory data of the N group (non-vertebral fracture) and F group (asymptomatic vertebral fractures).

	All Participants	N Group	F Group	*p* Value
	(N = 217)	(N = 195)	(N = 22)	
Male/female sex	89/128	82/113	7/15	0.355
Age (years)	64.9 ± 7.9	64.8 ± 7.8	66.1 ± 7.9	0.322
Height (cm)	158.6 ± 8.6	158.6 ± 8.3	158.6 ± 9.7	0.979
Weight (kg)	60.4 ± 11.1	60.1 ± 10.9	63.7 ± 11.8	0.223
BMI (kg/m^2^)	23.9 ± 3.4	23.8 ± 3.4	25.1 ± 2.9	0.031 *
Waist (cm)	80.4 ± 8.9	80.1 ± 8.9	83.0 ± 8.7	0.137
Hip (cm)	92.3 ± 6.1	92.1 ± 6.2	94.3 ± 5.0	0.079
BF%	29.5 ± 7.3	29.2 ± 7.1	33.0 ± 7.9	0.018 *
YAM (%)	80.9 ± 15.2	80.9 ± 15.2	80.3 ± 15.9	0.898
T score	0.3 ± 1.3	0.3 ± 1.3	0.3 ± 1.3	0.988
Z score	−1.7 ± 1.4	−1.7 ± 1.4	−1.8 ± 1.4	0.858
DM	12	11	1	0.831
GNRE	109.7 ± 7.3	109.4 ± 7.3	112.2 ± 6.7	0.087
SMI (kg/m^2^)	6.8 ± 1.0	6.8 ± 1.0	7.1 ± 1.0	0.357
Grip strength (kg)	28.9 ± 9.0	28.9 ± 9.0	29.2 ± 9.5	0.861
Back muscle strength (kg)	81.1 ± 32.0	80.9 ± 32.0	82.7 ± 32.4	0.875
Laboratory data				
Serum albumin level (g/dL)	4.4 ± 0.2	4.4 ± 0.2	4.4 ± 0.2	0.902
Total cholesterol level (mg/dL)	207.6 ± 34.5	207.5 ± 34.8	208.5 ± 31.0	0.989
TG level (mg/dL)	96.4 ± 58.8	96.4 ± 59.6	96.4 ± 50.3	0.940
CRP level (mg/dL)	0.1 ± 0.4	0.1 ± 0.4	0.1 ± 0.2	0.313

BMI: body mass index, BF%: body fat percentage, SMI: skeletal muscle mass index, DM: diabetes mellitus, *: *p* < 0.05.

**Table 2:** Radiographic parameters between the N and F groups.

	All Participants	N Group	F Group	*p* Value
	(N = 217)	(N = 195)	(N = 22)	
C2S	12.3 ± 9.2	12.2 ± 9.0	12.9 ± 8.4	0.794
CL	−7.6 ± 12.2	−7.6 ± 12.2	−7.9 ± 12.5	0.630
cSVA	25.0 ± 12.8	25.0 ± 13.0	25.5 ± 10.7	0.691
CPA	16.0 ± 9.0	16.3 ± 9.1	15.0 ± 7.8	0.428
CTPA	2.4 ± 2.6	2.4 ± 2.7	2.6 ± 1.1	0.510
T1S	21.4 ± 11.8	21.2 ± 11.7	23.2 ± 10.2	0.769
TK	23.0 ± 11.3	22.5 ± 10.7	26.4 ± 11.4	0.309
TLK	7.5 ± 11.8	7.1 ± 11.6	10.6 ± 13.3	0.347
TPA	13.7 ± 8.7	13.9 ± 8.7	13.4 ± 9.0	0.722
L4S1	−37.5 ± 14.3	−37.4 ± 13.4	−39.9 ± 13.8	0.662
LL	−44.0 ± 16.6	−44.0 ± 15.6	−46.0 ± 14.3	0.664
PI	49.1 ± 15.9	49.4 ± 15.1	50.1 ± 12.2	0.795
PT	18.6 ± 9.3	18.9 ± 9.3	18.3 ± 8.3	0.709
SS	30.6 ± 11.5	30.7 ± 11.0	31.8 ± 9.8	0.664
PI-LL	5.2 ± 11.7	5.6 ± 12.2	4.1 ± 9.7	0.941
KOA	65/149	54/138	11/11	0.035 *
KL gade0.1	149	138	11	
KL gade2.3.4	65	54	11	

C2S: C2 slope, CL: C2–7 lordosis, cSVA: C2–7 SVA, CPA: C2 pelvic angle, CTPA: C2–T1 pelvic angle, T1S: T1 slope, TK: thoracic kyphosis, TLK: thoracolumbar kyphosis, TPA: T1 pelvic angle, L4S1: L4−S1 lordosis, LL: lumbar lordosis, PI: pelvic incidence, PT: pelvic tilt, SS: sacral slope, PI-LL: pelvic incidence–lumbar lordosis, KOA: knee osteoarthritis, KL: Kellgren–Lawrence grade, *: *p* < 0.05.

**Table 3:** Risk factors of asymptomatic vertebral fractures in middle-aged and elderly adults based on the logistic regression analysis.

	B	SE	Wald	df	*p* Value	Exp (B)	95% Confidence Interval
Sex	0.601	0.683	0.775	1	0.379	1.824	0.479–6.953
BMI	1.128	0.527	4.583	1	0.032 *	3.091	1.100–8.684
BF%	−3.703	0.682	0.007	1	0.931	0.943	0.247–3.592

BF%: body fat percentage, *: *p* < 0.05.

**Table 4:** Quality of life and pain scale score between the N and F groups.

	All Participants	N Group	F Group	*p* Value
	(N = 217)	(N = 195)	(N = 22)	
Low back pain VAS score	16.1 ± 19.0	16.4 ± 19.1	14.3 ± 17.5	0.676
Leg pain VAS score	14.9 ± 21.8	14.9 ± 21.9	15.3 ± 20.3	0.421
Knee pain VAS score	14.5 ± 18.0	13.5 ± 18.8	23.4 ± 19.7	0.014 *
SF-36 PCS score	47.9 ± 10.5	48.1 ± 10.6	46.6 ± 8.9	0.346
SF-36 MCS score	51.8 ± 8.4	51.7 ± 8.5	52.4 ± 7.3	0.727
SF-36 RCS score	51.1 ± 9.8	51.4 ± 9.4	48.5 ± 12.8	0.432
EQ5D-5L score	0.9 ± 0.1	0.8 ± 0.1	0.9 ± 0.1	0.169
PainDETECT score	4.2 ± 4.4	4.1 ± 4.4	5.7 ± 4.0	0.043 *

VAS: visual analog scale, *: *p* < 0.05.

## Data Availability

The datasets generated and/or analyzed during the current study are available from the corresponding author upon reasonable request.

## Abbreviations

The following abbreviations are used in this manuscript:

BF%	Body fat percentage
BIA	Bioelectrical impedance analysis
BMAT	Bone marrow adipose tissue
BMD	Bone mineral density
BMI	Body mass index
CL	C2–7 lordosis
CPA	C2 pelvic angle
cSVA	C2–7 SVA
C2S	C2 slope
CTPA	C2–T1 pelvic angle
DM	diabetes mellitus
K-L	Kellgren-Lawrence grade
KOA	Knee osteoarthritis
LL	Lumbar lordosis
L4S1	L4–S1 lordosis
PI	Pelvic incidence
PI-LL	Pelvic incidence–lumbar lordosis
PT	Pelvic tilt
QOL	quality of life
SMI	skeletal muscle mass index
SQ	Genant’s semiquantitative
SS	Sacral slope
TK	Thoracic kyphosis
TLK	Thoracolumbar kyphosis
TPA	T1 pelvic angle
T1S	T1 slope
VAS	Visual Analog Scale
VF	Vertebral fracture
